# Element-specific investigations of ultrafast dynamics in photoexcited Cu_2_ZnSnS_4_ nanoparticles in solution

**DOI:** 10.1063/4.0000055

**Published:** 2021-04-07

**Authors:** Christian Rein, Jens Uhlig, David Carrasco-Busturia, Khadijeh Khalili, Anders S. Gertsen, Asbjørn Moltke, Xiaoyi Zhang, Tetsuo Katayama, Juan Maria García Lastra, Martin Meedom Nielsen, Shin-Ichi Adachi, Kristoffer Haldrup, Jens Wenzel Andreasen

**Affiliations:** 1 Department of Energy Conversion and Storage, Technical University of Denmark, Fysikvej, 2800 Kgs., Lyngby, Denmark; 2 NanoLund and Chemical Physics, Lund University, Box 124, 22100 Lund, Sweden; 3 X-ray Science Division, Argonne National Laboratory, Lemont, Illinois 60439, USA; 4 Japan Synchrotron Radiation Research Institute, 1-1-1 Kouto, Sayo, Hyogo, 679-5198, Japan, and RIKEN Spring-8 Center, 1-1-1 Kouto, Sayo, Hyogo, 679-5148, Japan; 5 Department of Physics, Technical University of Denmark, Fysikvej, 2800 Kgs., Lyngby, Denmark; 6 Institute of Materials Structure Science, High Energy Accelerator Research Organization (KEK), 1-1 Oho, Tsukuba, Ibaraki, 305-0801, Japan and Department of Materials Structure Science, School of High Energy Accelerator Science, The Graduate University for Advanced Studies, 1-1, Oho, Tsukuba, Ibaraki, 305-0801, Japan

## Abstract

Ultrafast, light-induced dynamics in copper–zinc–tin–sulfide (CZTS) photovoltaic nanoparticles are investigated through a combination of optical and x-ray transient absorption spectroscopy. Laser-pump, x-ray-probe spectroscopy on a colloidal CZTS nanoparticle ink yields element-specificity, which reveals a rapid photo-induced shift of electron density away from Cu-sites, affecting the molecular orbital occupation and structure of CZTS. We observe the formation of a stable charge-separated and thermally excited structure, which persists for nanoseconds and involves an increased charge density at the Zn sites. Combined with density functional theory calculations, the results provide new insight into the structural and electronic dynamics of CZTS absorbers for solar cells.

## INTRODUCTION

The quaternary compound Cu_2_ZnSnS_4_ (CZTS) crystallizing in the kesterite structure^1^ has been the subject of research as a potential thin-film photovoltaic absorber for more than ten years. The material is highly attractive for solar cell devices because it only contains earth abundant and nontoxic elements and could thus be scaled to the terawatt level. In contrast to silicon, CZTS is a direct bandgap (Eg = 1.5 eV) semiconductor, which allows for efficient thin (<1 *μ*m) absorber layers and, consequently, significantly less material use than for the typical silicon solar cell on the market today. The attained power conversion efficiency has, to date, not exceeded the 12.6% demonstrated in 2014 by Wang *et al.*^2^ for selenized CZTS despite its favorable properties and extensive research since. This is still far below similar thin film absorber materials like the CIGSe class of materials (22.9%)^3^ and much below the theoretical Shockley–Queisser limit of about 31%.^2^ Selenized CZTS requires processing temperatures of 5–600 °C, whereas the pure sulfur version can be processed at room temperature,^4^ which makes the manufacturing of these solar cells less energy intensive and thus more cost efficient.

**FIG. 1.**
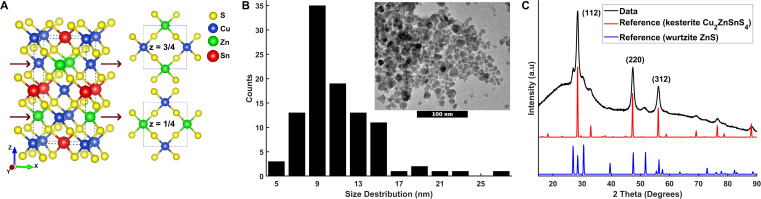
(a) Unit cell (5.43 Å × 5.43 Å × 10.84 Å) of CZTS showing sulfur (yellow), copper (blue), zinc (green), and tin (red) based on crystallographic reference data (ICSD 239686). (b) Histogram size analysis of the CZTS nanoparticles shown in the TEM image (insert). 100 nm scale bar. (c) XRD (Cu Kα radiation) pattern of CZTS nanoparticles, as well as the reference CZTS pattern (ICSD crystal structure: 239686) and reference wurtzite-ZnS pattern (ICSD crystal structure: 67453).

The reasons for the shortcomings in performance are likely manifold and presumably related to the complex crystal chemistry of CZTS including a high concentration of substitutional defects and elemental disorder.^5^ The synthesis of phase pure absorber layers has proven difficult with a high propensity for the formation of secondary phases that are inactive at best, but may be downright detrimental at worst, as is the case for CuS, which may short-circuit the photovoltaic device. Other mechanisms of failure include charge recombination at grain boundaries or at the interface to electrodes, leading to reduced mobility and poor charge transfer out of the absorber layer. Amongst all of these failure mechanisms, there is little consensus about which are the most detrimental,^5^ or in some cases, whether their effect is beneficial, e.g., influence of the Cu/Zn disorder in the z = 1/4 and z = 3/4 planes, see Fig. 1(a).^6–8^ It is difficult to determine how to address the shortcomings of CZTS photovoltaics without a fundamental understanding of how charges are generated and transported in the material.

The charge carrier dynamics of photo-excited CZTS thin films and single crystals have previously been studied using transient reflectometry (VIS/IR/THz) and time-resolved photoluminescence methods. In a series of studies, Phuong *et al.*^9–11^ determined via time-resolved reflectometry that carrier relaxation and trapping takes place on single-picosecond time scales and is dependent on (excess) excitation energy.^10^ Photoluminescence experiments indicated that carrier recombination is strongly temperature dependent and at room temperature takes place primarily on ∼100 ps time scales but with a significant component with nanosecond (ns) decay dynamics in agreement with Gokmen *et al.*, who reported lifetimes in the ns time range following excitation at 532 nm.^7^ In a very recent study, Li *et al.*^12^ utilized 370 nm excitation combined with near-infrared transient reflectometry to determine the timescale(s) for carrier relaxation and trapping to be in the 1–2 ps regime with recombination on ∼50 ps time scales. These observations of complicated carrier dynamics following photo-excitation are generally interpreted as consequences of Cu/Zn disorder^8^ and so-called “band-tailing” with short-range spatial variations in the valence- and conduction band (CB) energy landscapes leading to charge carrier trapping.^13^

Density functional theory (DFT) calculations published by Paier *et al.* suggest a model of the band structure around the bandgap E_g_.^14^ It shows the valence band (VB) being dominated by the antibonding hybrid Cu-3d/S-3p^*^ orbitals, whereas the bottom of the conduction band (CB) mainly consists of the antibonding hybrid Sn-5s/S-3p^*^ orbitals. The strong mixing of S-3p states into both the VB and the CB is suggested to be one of the reasons behind the high light absorption coefficient (10^4^) of CZTS^14^ as it overcomes the dipole selection rule of the transition. Based on the orbitals contributing to the valence band, the electronic state and local structure around the Cu atoms are thus of interest when trying to determine which structural properties govern the light absorption properties and excited-state properties of CZTS.

An effective method for probing the unoccupied electronic states, oxidation state, and geometry of CZTS is x-ray absorption spectroscopy (XAS) where x-ray-induced electronic excitations from highly localized core electronic levels (K-edge) are used to probe both the oxidation states for a specific element and the local structure around the absorbing nuclei. X-ray absorption near edge spectroscopy (XANES) contains information on the occupancy of electronic states at/near the probed element. Extended x-ray fine structure (EXAFS) occurring energetically well above (>10 eV) the XANES region is dominated by scattering contributions from local atomic geometry.^15^ XANES analysis has been used previously to study CZTS band structures,^16–18^ whereas EXAFS analysis has provided information on the local structure of CZTS.^19–22^

The work presented here aims to contribute to the understanding of charge generation and transport in CZTS via ultrafast time resolved x-ray absorption spectroscopy at the Cu and Zn absorption edges of near phase-pure nano-crystalline CZTS. Time-resolved XANES and EXAFS are used to track the electronic and structural effects as a function of time, an approach previously demonstrated for perovskite nanoparticles.^23^ We demonstrate that time-resolved x-ray absorption spectroscopy can distinguish the elements participating in the photovoltaic mechanisms in CZTS materials and provide information on how excitation modifies the electronic and nuclear structure.

## METHODS

### Sample preparation

CZTS nanoparticles stabilized by oleylamine were prepared by “hot injection” synthesis following previously established methods^24^ (details provided in the supplementary material, S1). The resulting nanoparticle powders were washed with isopropanol and dried. Nanoparticle solutions for characterization and experiments were prepared by adding 2 mg/ml nanoparticle powder to anhydrous toluene (Sigma Aldrich), after which the solution was sonicated for a minimum of one hour and filtered/decanted. The filtered sample solution had a concentration of 1 mg/ml as determined by UV-vis optical absorption.

### Electron microscopy and x-ray characterization

Toluene solutions of the nanoparticle samples were prepared and dropcast on TEM grids and Si substrates. TEM characterization [Fig. 1(b)] of the nanoparticle size and morphology was performed using a JEOL 2100 TEM with an accelerating voltage of 200 kV. The sample composition (S4) was characterized using a TM3000 (Hitachi) scanning electron microscope with integrated energy dispersive x-ray spectroscopy (EDX). X-ray diffraction was performed using a Rigaku “SmartLab” setup with a Cu K_α_ source to determine the CZTS nanoparticle crystal phases and average crystallite sizes [Fig. 1(c)].

### Optical spectroscopy

Raman spectroscopy was performed at 532 and 785 nm using a Renishaw InVia spectrometer to characterize sample disorder. The bandgap energy (E_g_) of the synthesized nanoparticles was estimated with optical spectroscopy, using a Jasco V-730BIO Spectrophotometer.

Transient Optical Absorption Spectroscopy (TOAS) was carried out using a Ti:Sapphire amplified laser system (Spitfire XP Pro, Spectra Physics) operating at a 1 kHz repetition rate, producing 0.05 ps pulses centered at 796 nm. The pump beam was created by frequency doubling the fundamental beam to excite the sample at 400 nm with 2 mJ/cm^2^/pulse. A supercontinuum white-light was used as probe, generated by focusing a NIR signal from a TOPAS C into a 5 mm sapphire plate. The desired timing between excitation and probe pulses was achieved using a computer controlled delay line (Aerotech, 10 ns).

### Time-resolved x-ray absorption spectroscopy (XAS)

Time-resolved XAS data with time delays from ∼0.1 ps to 20 ps were collected at the BL3 beamline^25^ at the SPring-8 Angstrom Compact Free Electron LAser (SACLA) facility^26^ using a well-established laser-pump/x-ray probe scheme at 30 Hz. The sample was excited at 15 Hz with 60 femtosecond laser pulses centered at 400 nm/3.1 eV with a fluence of ∼40 mJ/cm^2^/pulse. The timing diagnostics,^27^ which measures the timing jitter between laser and x-ray pulses, were used to suppress the timing drift during the beamtime. The sample response as a function of incident fluence was measured and all results presented here are in the linear regime. The x-ray probe pulses arrived at 30 Hz, with the beam size focused to 50 *μ*m with Be compound refractive lenses^28^ in a near collinear geometry. The energy of the incoming x-ray pulses was selected with a Si(111) monochromator^28^ without simultaneous scanning of the X-ray Free Electron Laser (XFEL) undulators, limiting the probe energy range to < 50 eV, the Self-Amplified Spontaneous Emission (SASE) bandwidth at both the Cu and the Zn absorption edge.

Total fluorescence yield detection was used, with photodiodes mounted in the horizontal plane at 90° to the beam propagation direction, utilizing the corresponding Z-1 foils with three absorption length thickness to suppress elastic scattering contributions to the detected signal.

The sample was delivered to the interaction region in a recycled 150 *μ*m thick round jet inside an experimental chamber with a helium atmosphere.^29^ The total sample volume was ∼50 ml, continuously magnetically stirred and frequently topped-up with solvent to compensate for evaporation. The sample condition was continuously monitored via the intensity and shape of the unpumped XAS signal and regularly exchanged before the ground state XANES showed any difference in shape.

Time-resolved XAS data at time delays from ca. −100 ps to 5 ns were collected at beamline 11-ID-D at the Advanced Photon Source (APS) of Argonne National Laboratory.^30^ The 400 nm, 1.6 ps FWHM (Full Width Half Maximum) laser pump pulse (12 mJ/cm^2^/pulse at sample position) was the second harmonic output of a Ti:Sapphire regenerative amplified laser operating at 10 kHz repetition rate. The x-ray probe pulses were derived from electron bunches extracted from the storage ring with 79 ps fwhm and 6.5 MHz repetition rate. The energy of the incoming x-ray pulses was selected with a Si(111) monochromator with simultaneous scanning of the undulator gap, allowing an extended scan range of several 100 eV.

Total fluorescence yield detection was used, with photodiodes mounted in the horizontal plane at 90° to the beam propagation direction and utilizing Z-1 filters of 3 absorption length thickness as well as Soller slits to suppress elastic scattering contributions to the detected signal.

The sample was delivered to the interaction region in a 650 *μ*m round jet inside a compact chamber with a helium atmosphere. The total sample volume was ∼50 ml with the sample container being continuously magnetically stirred and cooled. The liquid sample in the reservoir was constantly bubbled with solvent saturated N_2_ gas to maintain an O_2_ free environment and to replenish the solvent lost by evaporation. The sample condition was continuously monitored via the intensity and shape of the unpumped XAS signal and the sample was replaced when a change of the ground state XANES was visible.

For both TR-XAS experiments at SACLA and APS, the energy was calibrated by setting the first peak of the derivative absorption spectra of metallic Cu- and Zn-foils to 8979 and 9659 eV, respectively. The details of the data processing protocol are presented in the supplementary material (S2).

### Density functional theory (DFT) simulations

Density functional theory simulations were carried out within the VASP^31^ framework to explore the connections between electronic and structural degrees of freedom following photo-excitation. Kohn–Sham valence states (Cu-3d, Cu-4p, Zn-3d, Zn-4p, Sn-5s, Sn-5p S-3s, and S-3p) were expanded with a plane wave basis set using a kinetic energy cutoff of 400 eV. Projector-augmented wave (PAW) pseudopotentials^32,33^ were used to describe core electrons.

The atomic positions of a 2 × 2 × 1 supercell of kesterite-structured CZTS consisting of 64 atoms were optimized with the hybrid exchange-correlation functional of Heyd–Scuseria–Ernzerhof (HSE06).^34^ The Brillouin zone was sampled using a 2 × 2 × 2 Monkhorst–Pack k-point mesh.^35^ Previous studies have shown that the aforementioned supercell size and k-point mesh are appropriate for the electronic structure investigation of defects in kesterite.^36,37^ The atomic positions were relaxed until the forces per atom converged to less than 0.02 eV Å^−1^. The break condition for the electronic self-consistent loop was set to 1 × 10^−5 ^eV, and a bandgap of 1.4792 eV was obtained. Full geometry optimization (including lattice vectors) with the Perdew–Burke–Ernzerhof (PBE) exchange-correlation functional^38^ and Hubbard *U* correction for the Cu-d states^39^ was performed with a *U* value of 6.0 eV in correspondence with previous studies.^40^ A bandgap of 0.6566 eV was obtained, which corresponds to an offset of 0.82 eV with respect to the above HSE calculation. All possible excitations of one electron from the valence to the conduction band that fall within an energy difference no greater than 3.1 − 0.82 = 2.3 eV were considered through a Delta Self-Consistent Field Δ(SCF) approach, in which the excited state is calculated by enforcing the corresponding non-Aufbau occupation of the bands.^41^ In order to get the relaxed geometries for the excited states, the same non-Aufbau band occupation numbers are kept constant during subsequent SCF cycles of the geometry optimization, in which the volume and shape of the unit cell are kept fixed. These excitations induce a slight distortion of the atomic positions with respect to the ground state, as will be commented in the Results section.

### EXAFS simulations

To benchmark the DFT calculations against the time-resolved data, the DFT-derived structural motifs were used to calculate the (difference) Extended X-ray Absorption Fine Structure (EXAFS). This was done using the FEFF6 software package^42^ and using the muffin-tin approximation.^43^ The structures (ground- and excited state) calculated from DFT were kept constant, with the Debye–Waller factor, E_0_ and the amplitude reduction factor allowed to vary in the fitting procedure. Further details can be found in the supplementary material (S3).

## RESULTS

### Sample characterization

Figure 1(b) shows a representative TEM image of the synthesized nanoparticles and a histogram of particle sizes measured in a series of such images. We find the nanoparticles to be irregular with faceted morphologies and with a mean particle size of 11 nm ± 4 nm. EDX analysis (S4) indicates a Cu/Sn ratio of 2.00 and a Zn/(Cu + Sn) ratio of 0.77.

Figure 1(c) shows an x-ray diffraction pattern of the nanoparticle solution dropcast on a silicon substrate. Included in Fig. 1(c) are reference x-ray diffraction patterns for kesterite CZTS and for wurtzite ZnS from the Inorganic Crystal Structure Database (ICSD). In the diffraction pattern of the drop-cast nanoparticle solution, all the major peaks of the CZTS reference can be identified, as can several of the most intense diffraction peaks of the ZnS secondary phase. From the Scherrer equation applied to the width of the 220 kesterite peak (S5) we find a crystallite domain size of ∼10 nm in good agreement with the TEM result. Similar results were obtained for CZTS nanoparticles used for TR-XAS with ps–ns resolution (S6).

**FIG. 2.**
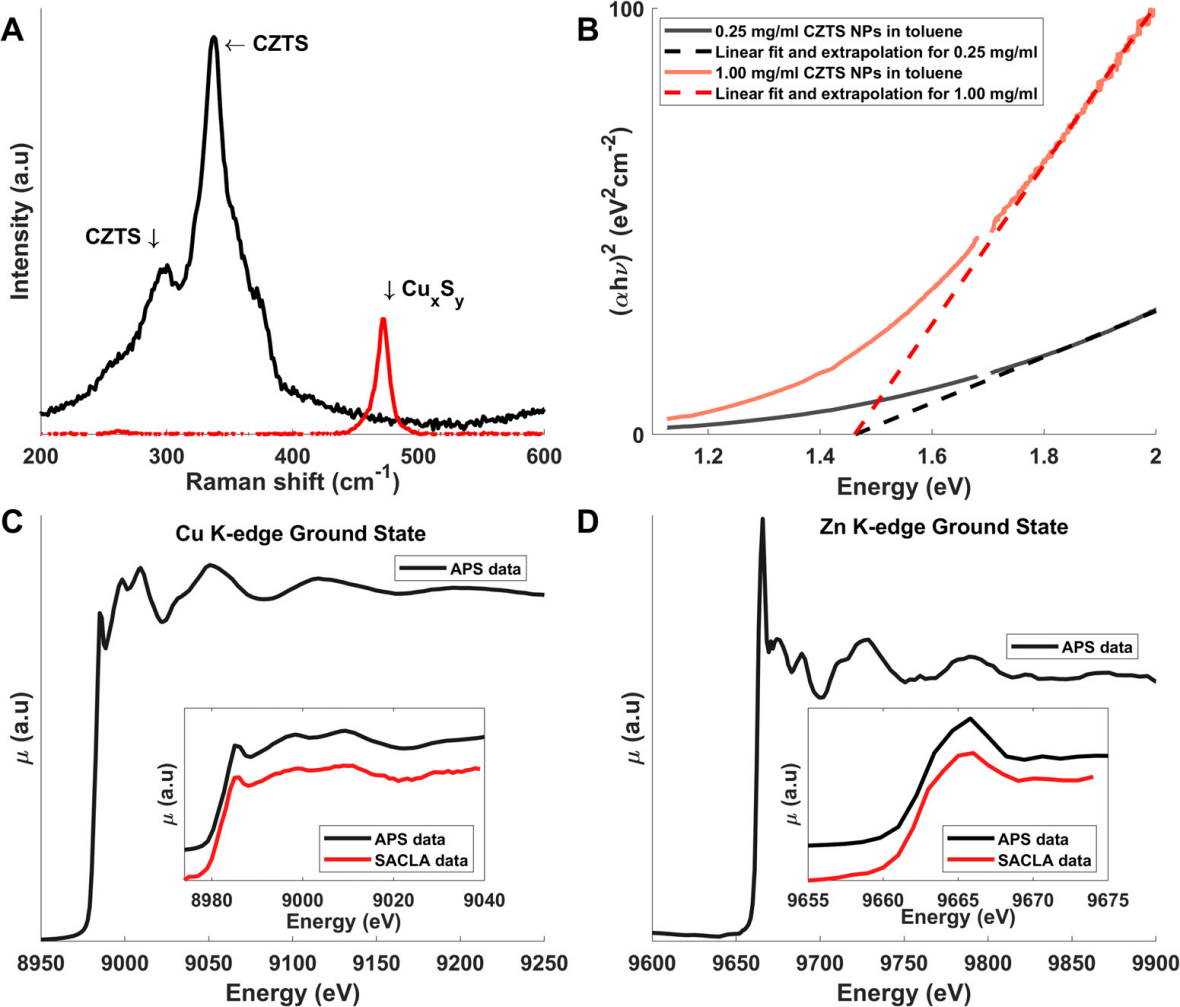
(a) Raman spectrum (black) of the CZTS sample acquired at λ = 532 nm. Shown in red is the reference spectrum for Cu_x_S_y_. (b) Tauc-plot showing the (re-scaled) absorbance as a function of photon energy for two sample concentrations. Linear extrapolation of the high-energy part of the spectrum indicates E_g_ = 1.45 eV. (c) and (d) XAS spectra at the Cu and Zn K-shell absorption edges as acquired at the APS synchrotron with XANES region comparison for both APS and SACLA data (details in S9).

Figure 2(a) shows the Raman spectrum obtained at a wavelength of 532 nm. A strong peak at the known^44^ CZTS A-mode frequency at 337 cm^−1^ is observed, and an absence of intensity at the Cu_x_S_y_ vibrational mode at 475 cm^−1^ is noted.^4^ A full analysis^45^ of the Raman spectrum observed at 785 nm excitation (S7) indicates a Cu-Zn ordering parameter Q = 1.41, where fully ordered CZTS has been shown to have Q = 4.15 and fully disordered CZTS exhibits Q = 0.77. In terms of bandgap, Tauc-analysis of the absorption spectrum [Fig. 2(b) and S8] indicates a direct bandgap with an energy of E_g_ = 1.45 eV, quite close to the E_g_ = 1.5 eV previously reported for bulk-like CZTS thin-films. Figures 2(c) and 2(d) show steady state x-ray absorption spectra at the Cu and Zn absorption edges at 8983.5 eV and 9662.5 eV, respectively, with no pre-peak features on either edge. As discussed in more detail below, this is consistent with previous assignment of Cu^+1^ and Zn^+2^ oxidation states and 3d^10^ electron configuration at both sites in comparable CZTS references (S9).

### Time-resolved measurements

#### Optical

**FIG. 3.**
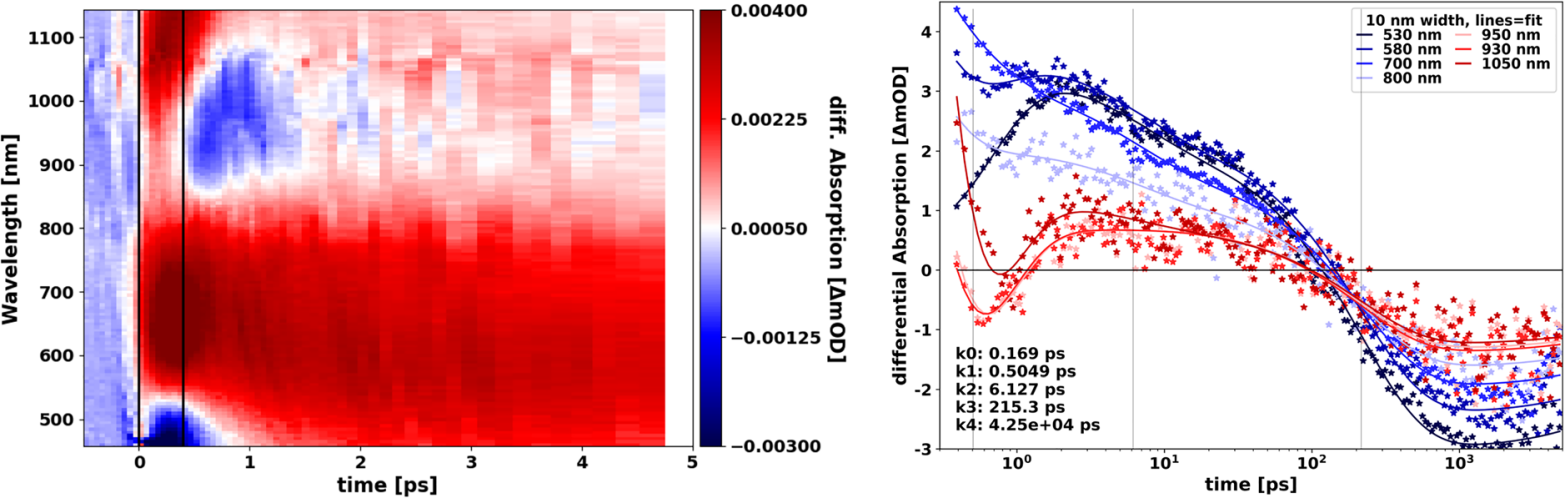
(a) TOAS difference signal ΔA(λ, t). The positive signal corresponds to increased absorption following photo-excitation. (b) Kinetic evolution of the TOAS signal at selected wavelength with fit based on a global analysis between 0.45 ps and 5 ns (S10).

Turning first to Transient Optical Absorption Spectroscopy (TOAS), Fig. 3(a) shows the change in sample absorption, Δ*A*(*λ*, *t*), following 400 nm photoexcitation of the CZTS nanoparticle solution. In agreement with transient reflectometry measurements,^9,12^ Δ*A*(*λ*, *t*) shows quite complex dynamics with evolution on several different time scales and with a strong wavelength dependence despite the very simple absorption spectrum [Fig. 2(b)]. To determine the key time constants from the complex evolution of the spectra shown in Fig. 3(a), the full dataset was fitted in a Global Analysis framework with five time constants (S10 for details). Figure 3(b) shows the associated fits to the experimental data at selected wavelengths with the lower left legend indicating the best-fit time constants.

For short time delays [<0.4 ps, thin black line in Fig. 3(a)], the most prominent features are well-defined Excited-State Absorption features (ESA, positive signal) at 530–800 nm and at >1000 nm as well as a bleach (negative signal) at <520 nm. These features appear promptly, decay with a time constant of 0.50 ps, and are replaced by a broader ESA (500–800 nm) and a narrow bleach at 850–1050 nm. On a timescale of a few picoseconds, the long-wavelength bleach disappears, the ESA shifts to shorter wavelengths, and a species with very broad ESA and a lifetime of a few hundred ps is formed. This difference signal component is replaced by a negative signal reminiscent of the inverse of the ground state absorption spectrum. We note that for time delays <0.15 ps, the TOAS data are influenced by solvent dynamics and measurement artifacts, which in turn affects the determination of the initial grow-in time constant (0.17 ps), which should thus be interpreted with some care.

#### Time-resolved X-ray Spectroscopy results

**FIG. 4.**
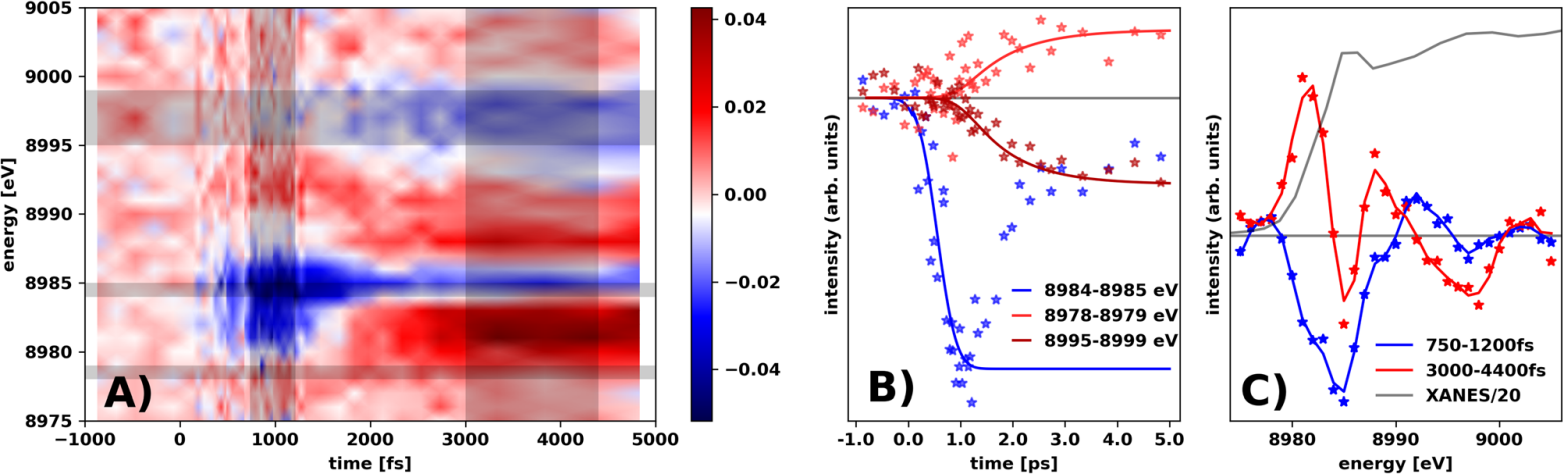
(a) Δμ(Ε, t) at the Cu K-edge. Black-shaded regions indicate the temporal and energy profiles shown in 4B and 4 C. (b) Time evolution of the difference signal at three characteristic energies; full lines are the kinetic fits described in the main text. (c) Spectral shape of Δμ(Ε, t) in two characteristic time ranges, with interpolated lines to guide the eye.

Figure 4(a) shows the change in the Cu K-edge x-ray absorption Δ*μ*(*Ε, t*) on single-picosecond time scales. Immediately following photo-excitation, the transient signal is dominated by a strong decrease in absorption at 8985 eV. On a few-picosecond timescale, Fig. 4(a) further shows how this immediate (sub-picosecond) bleach is replaced by a more complex difference signal. Figure 4(b) shows the time evolution of the difference signal at selected energies (horizontal gray bars) and Fig. 4(c) shows Δ*μ*(*Ε*) at time scales where each of the two characteristic difference signal components dominates [Fig. 4(a), vertical gray bars] as well as the ground-state XANES spectrum for reference. In comparison to the shown ground state absorption, we note that both the immediate bleach and the following more complex difference signal have dominant features at the edge. To estimate the time constant of these two difference signal components, the data in three spectral regions were individually fitted by exponential grow-ins with time constants τ_1–3_ and broadened with an Instrument Response Function (IRF, see the supplementary material for details and full fit results) and allowing the onset of the exponential grow-in to vary freely. Figure 4(b) shows the data and the fits, with the 8985 eV signal exhibiting a grow-in time τ_1_ < 0.3 ps. The data traces at higher and lower energies are observed to have an onset delayed by 0.5(2) ps compared to the 8985 eV signal, with grow-in times τ_2,3_ = 0.9(1) ps. A Singular Value Decomposition (SVD) confirms that two components are sufficient to describe the acquired difference signal and highlights how the fast signal is well represented by a simple shift of the edge, but with the slower signal exhibiting more complex features (S11).

**FIG. 5.**
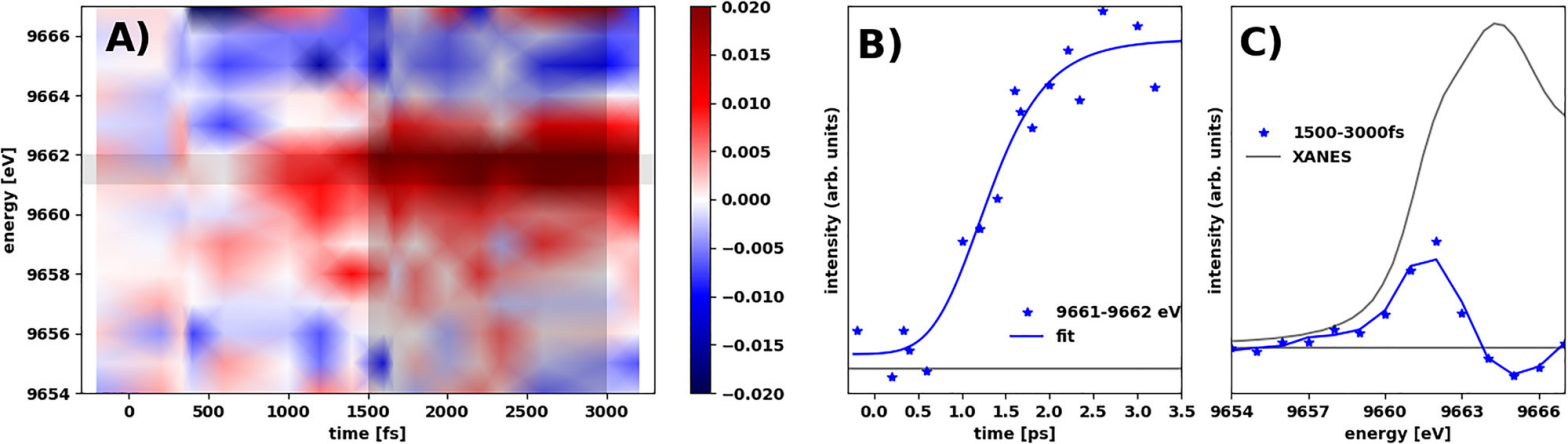
(a) Δμ(Ε, t) at the Zn K-edge interpolated using Gouraud-type shading. Black-shaded regions indicate the temporal and energy profiles shown in (b) and (c). (b) Time evolution of the difference signal at 9661 e; the full line is the kinetic fit described in the main text. (c) The full absorption spectrum (gray line) and averaged spectral shape of Δμ(Ε, t) at t = 1.5–3 ps.

Figure 5 shows the differential absorption Δ*μ*(*Ε*, *t*) at the Zn edge. The difference signal intensity is significantly lower than at the Cu edge, and the difference signal in the entire investigated time range is well described by a single component, an excited-state edge shift toward lower energies (S12). Fitting the rise in the signal shown in Fig. 5(b) with the same model as applied for the difference signal at the Cu edge, we find that the time evolution is very well described by a model with the same delayed onset [0.5(2) ps compared to onset of the fast Cu signal] and grow-in time [τ_Zn_ = 0.9(1) ps] as the slow component at the Cu edge.

**FIG. 6.**
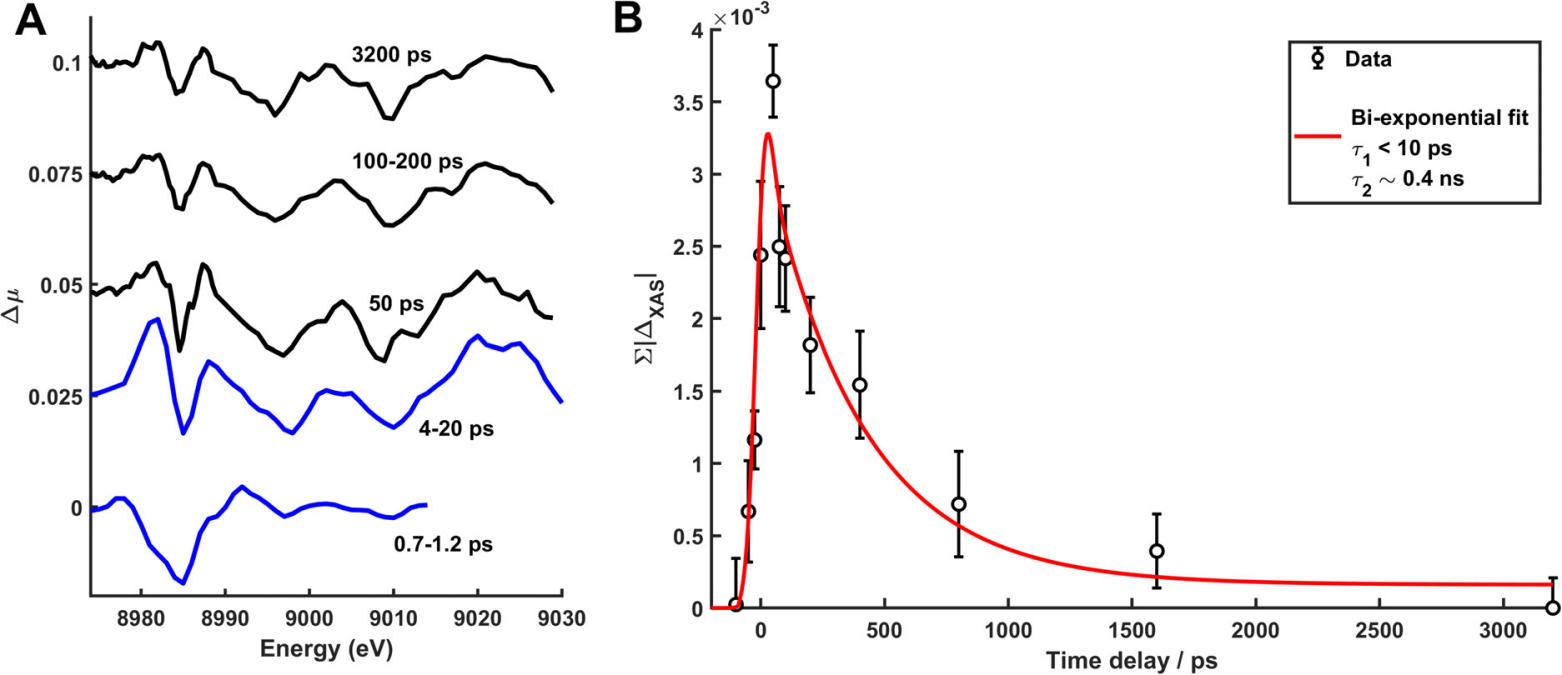
(a) Δμ-difference signals (second order Savitzky–Golay filtered) for Cu-edge XAS offset by 0.025 between each difference signal. Black and blue lines indicate data taken at APS and SACLA, respectively. At SACLA, only data <9015 eV were acquired at the shortest time delays. (b) Time evolution of absolute sum of the Cu-edge Δμ difference signal in the range of 8929–9029 eV (black points) and a bi-exponential fit (red line).

Extending the short-time measurements of Δ*μ*(*Ε*, *t*) done at SACLA, Fig. 6(a) shows the Cu K-edge difference spectra as measured at APS in the time range from 0 ps to several ns and with a time resolution of ∼100 ps (data for the full set of time delays shown in S13). Also shown are the two characteristic difference spectra acquired at SACLA at *t *=* *0.7–1.2 ps (black) and at 4–20 ps (red). Very little change in signal shape is evident from *t *=* *4 ps onwards (details in S14), but the magnitude of the difference signal is observed to decrease. Characterizing this decrease in further detail, Fig. 6(b) shows the absolute sum of the difference signals acquired at APS in the time range from *t* = −50 ps to *t *=* *3.2 ns (black points). We observe that the temporal evolution of the difference signal is well described by an immediate (on APS time scales) grow-in of the difference signal, followed by a bi-exponential decay (red fit line) with time constants τ_1_ < 10 ps and τ_2_ ∼ 0.4 ns, where the confidence interval on the long time constant is rather wide, 95%CI = 0.2–3.5 ns. We note, however, that the difference signal shown for t = 3.2 ns in Fig. 6(a) clearly shows the same features as for the shorter time delays, ruling out the lower ranges of the confidence interval. A similar dynamic could be observed on a sample from a different synthesis batch despite a lower signal-to-noise ratio (S15).

**FIG. 7.**
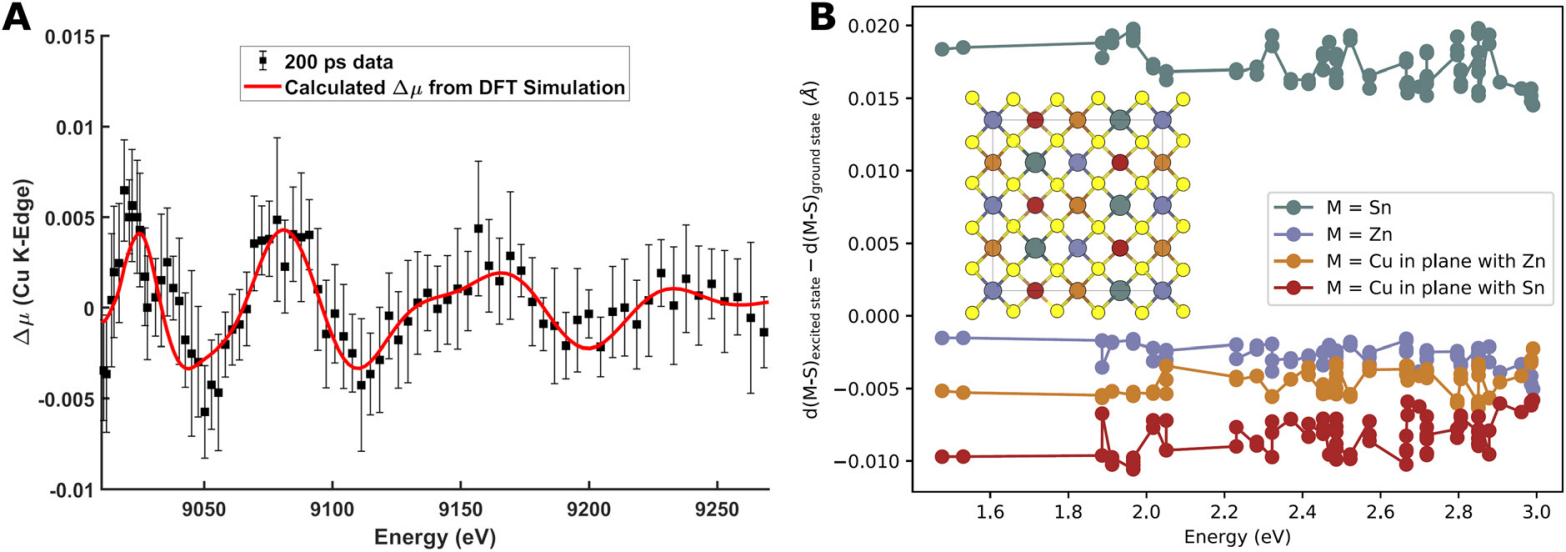
(a) Measured difference signal Δμ(E) at t = 200 ps (black points) above the Cu K edge. The red line shows the fit result calculated from the DFT results in panel B. (b) Representation of the 2^*^2^*^1 supercell used for the DFT calculations along with the metal-S bond length changes for each of the investigated excited states following excitation with ΔE = 1.5–3 eV.

Transient structural changes after photo-excitation were studied using differential x-ray absorption Δ*μ*(*Ε, t*) in the EXAFS regime at 200 ps time delay. Figure 7(a) shows the acquired EXAFS difference signal (black points) as well as a simulated EXAFS difference signal (red line) based on the DFT simulations of the ground- and excited state structures as discussed above. Figure 7(b) shows the excited-state equilibrated DFT results for metal-sulfur bonds as changes relative to the ground state structure and shown as a function of excitation energy. The structural changes are observed to be generally independent of this parameter, and a lengthening of the Sn-S bonds and a shortening of the Cu-S bonds are predicted following photo-excitation. These structural changes stems from the nature of the photo-excitations, where antibonding Cu_d_-S_p_ valence bands are emptied and antibonding Sn_s_-S_p_ conduction bands are filled. We note that to accurately reproduce the acquired difference signal shown in Fig. 7(a), an increase in 20% in the Debye–Waller factor for the excited-state structure was needed and that this contribution dominates the change in the EXAFS signal compared to the structural parameters.

### Discussion

Referring to the TEM and XRD measurements in Fig. 1, the CZTS nanoparticles measured here are predominantly ∼10 nm CZTS nanoparticles of kesterite structure. Although a small amount of wurtzite-structured material [presumable ZnS, Fig. 1(c)] was detected, no other secondary phases were observed. In particular, the presence of Cu_x_S_y_ material, detrimental for the function of a solar cell due to its metallic nature, could be excluded via Raman spectroscopy at λ = 532 nm [Fig. 2(a)]. As discussed in the introduction, Cu and Zn are often disordered at the z = 1/4 and z = 3/4 positions [Fig. 1(a)] as observed in high-performing CZTS solar cells.^8^ For the material investigated here, IR-Raman spectroscopy indicates a high degree of disorder (Q = 1.4) as estimated via the mode analysis presented in the supplementary material, Sec. IV. We measured a high absorbance (*μ* = 10^4^ cm^−1^) of the sample and an absorption spectrum monotonously decreasing with decreasing photon energy (S8), with a slope consistent with a bandgap of E_g_ = 1.45 eV (λ = 855 nm), again confirming CZTS as a promising material for solar energy harvesting applications.

Figure 2(c) shows the x-ray absorption spectrum acquired in the XANES region of the Cu K-edge. The position of the absorption edge (8983.5 eV) and the lack of pre-edge features indicate that the Cu atoms are in a + 1 oxidation state meaning in the 3d^10^ electron configuration. The Zn K-edge XAS spectrum in Fig. 2(d) indicates that the majority of the Zn atoms (which are involved in the formation of a second CB ca. 3 eV above the valence band^14^) are in the formal +2 oxidation state, corresponding also to a 3d^10^ closed-shell electronic configuration.

For the Cu atoms, TR-XAS show two clearly separated dynamical processes (Fig. 4). The bleach around 8985 eV of the edge region is the first process, and as can be seen in Figs. 4(a) and 4(b) (supported by the SVD, results in S11 in the supplementary material), this feature is formed very fast and is consistent with a shift of the absorption edge to higher binding energies (S16a). This observation of K-shell stabilization has also been observed in Cu complexes following photo-excitation^46–48^ and is interpreted as arising due to less screening from outer-shell electrons removed by the photo-excitation, i.e., a photo-oxidation of the involved copper atoms.

Following this immediate blue-shift of the Cu edge upon photo-excitation, a more complicated difference signal gradually develops. Referring to Fig. 4 and the related analysis, this second difference signal evolves on a timescale of single picoseconds and cannot be reproduced by a simple edge shift, but a preliminary analysis (SI, S16B) suggests that some electron (back-) transfer to Cu sites (a reduction process) combined with structural re-arrangements around the Cu ions may be involved. We note that this feature of the XANES difference signal, which we thus interpret as arising from electronic and structural relaxation, takes place on the same timescale as the blue-shift of the short-wavelength (500–750 nm) ESA feature in the TOAS data, consistent with a shift of the excited-state electron population to lower energies. Interestingly, the XANES difference signal observed at the Zn edge [Fig. 5(a)] develops on similar time scales and is consistent with an electron transfer to and structural changes around the Zn sites in the CZTS nanoparticles. Fitting the ps-scale Zn-edge APS data indicates a combination of thermal and structural effects (S17).

Based on this, we suggest, in agreement with Paier *et al.*,^14^ that the CZTS conduction band contains contributions from the Zn 3d orbitals, in particular, near the lower band edge, i.e., in the region of the so-called tail states suggested as key sites for the long-lived state(s).^13^

Turning next to the long term dynamics at the Cu edge, the constant shape of the difference signals measured at SACLA/APS and presented in Fig. 6 demonstrates that during the first few picoseconds, a stable excited-state structure has been established. This structure is well described by the DFT model introduced above in combination with significant nanoparticle heating, consistent with the release of electronic energy in the relaxation process described in the preceding paragraph.

The time evolution of the absolute magnitude of the difference signals measured at APS is well captured by a bi-exponential decay with an unresolved fast (<10 ps) decay component followed by a slower decay on a ns timescale. We associate the former with contributions from the strong bleach signal (Fig. 4) and the latter with the return to the ground state from the charge-separated excited state, in agreement with previous time-resolved optical studies.^7,10^ In comparison with the TOAS data shown in Fig. 3, the return to the ground state is significantly slower in the XANES data if we identify the broad ESA signal with the charge-separated state. As such, we cannot rule out that a main contribution to the difference signals shown in Figs. 4–6 at the ns timescale is of thermal origin, reflecting the cooling time of the nanoparticles^49^ and suggest that some of the reflectivity data discussed in the literature may also be of thermal rather than electronic origin.^50^ This thermal component of the XAFS signal would then be related to multi-photon excitation of the individual nanoparticles rather than being caused by the comparatively high energy of the exciting photons compared to the bandgap, as data acquired by Li *et al.*^12^ with 370 nm excitation do not show any components on time scales longer than a few hundred ps. Further experiments with excitation at both lower photon energies and fluences may serve to elucidate this observation of different time scales further.

## SUMMARY AND CONCLUSIONS

Based on the results and discussion presented above, we suggest the following chronology for the dynamics following 400 nm photo-excitation of CZTS nanoparticles: At the very shortest time scales, 0–0.3 ps, we observe a Cu K-edge difference signal indicating photo-oxidation (electron removal) from Cu atoms following photo-excitation. On slightly longer time scales (0.5–3 ps), we observe the evolution of a more complex difference signal at the Cu K-edge. This second difference signal is established on a timescale of ∼1 picosecond and preliminary analysis suggests that electron back-transfer to Cu sites combined with structural re-arrangements around the Cu ions may be involved. On the same time scales, the difference signal acquired at the Zn K-edge develops features also consistent with a reduction. We interpret the time evolution of the Cu and Zn edges to arise as a consequence of relaxation of the photo-excited electrons to the bottom of the CB, accompanied by structural changes due to the new electronic configuration (Fig. 7) and heat release to the lattice as the electronic system relaxes. This charge-separated high-temperature state of the CZTS nanoparticles persists for at least several hundreds of picoseconds before returning to the ground state. This is comparable to the lifetime of trap-assisted recombination (260 ps) observed for other Cu-poor/Zn-rich CZTSe with 5.7% solar cell efficiency excited by 400 nm^12^ when factoring in the cooling of hot carriers in CZTS.^9^

In conclusion, we here presented the synthesis and static and time-resolved characterization of CZTS nanoparticles supported by DFT calculations. By combining optical spectroscopy with time-resolved x-ray absorption spectroscopy, we achieve element-specificity and conclude that the photo-excited electrons originate from a valence band region arising at least partially from Cu-centered orbitals and that the photo-excited state involves structural and electronic changes at both the Cu and Zn sites, with the time evolution suggesting involvement of the Zn-centered orbitals in the low-energy part of the conduction band landscape.

## SUPPLEMENTARY MATERIAL

See the supplementary material for a description of the synthesis of the nanoparticles used in this study; a detailed description of the data reduction procedure applied to time resolved XAS data; description of fitting of simulated EXAFS data to experimental data; summaries of characterization of the nanoparticles by Energy Dispersive x-ray Spectroscopy, x-ray powder diffraction, Transmission Electron Microscopy, Raman spectroscopy, Ultraviolet-Visible light spectroscopy, and ground state x-ray absorption spectroscopy; a detailed description of the acquisition of transient optical absorption spectroscopy data and their analysis; and supplementary time-resolved x-ray absorption spectroscopy data, including analysis of absorption edge shifts at the Cu and Zn k-edge at various time delays, long time delay data, and additional data from a second batch of material.

## AUTHORS' CONTRIBUTIONS

Authors C. Rein and J. Uhlig contributed equally to this work.
